# Significant Improvement in Dynamic Visual Acuity after Cataract Surgery: A Promising Potential Parameter for Functional Vision

**DOI:** 10.1371/journal.pone.0115812

**Published:** 2014-12-26

**Authors:** Mingxin Ao, Xuemin Li, Chen Huang, Zhiqiang Hou, Weiqiang Qiu, Wei Wang

**Affiliations:** 1 Department of Ophthalmology, Peking University Third Hospital, Beijing, China; 2 Key Laboratory of Vision Loss and Restoration, Ministry of Education, Beijing, China; 3 Medical Research Center, Peking University Third Hospital, Beijing, China; Medical College of Soochow University, China

## Abstract

**Purpose:**

Dynamic visual acuity (DVA) is a relatively independent parameter for evaluating the ability to distinguish details of a moving target. The present study has been designed to discuss the extent to which age-related cataract impacts DVA in elderly individuals and to determine whether it could be restored after bilateral phacoemulsification combined with intraocular lens implantation surgery.

**Methods:**

Twenty-six elderly cataract patients scheduled for binocular cataract surgery and 30 elderly volunteers without cataract were enrolled in the study. DVA at 15, 30, 60 and 90 degree per second (dps) was assessed, and velocity-dependent visual acuity decreases between consecutive speed levels were calculated.

**Results:**

Compared with the control group, the patient group exhibited significantly worse DVA performance at all speed levels (p<0.001), and the decreases in velocity-dependent visual acuity were more serious in the patient group at the intervals of 0–15 dps (p<0.001), 15–30 dps (p = 0.007) and 30–60 dps (p = 0.008). Postoperatively, DVA performance at every speed level in the patient group clearly improved (p<0.001) and recovered to levels compatible to the control group. The decrease in visual acuity with increasing speed was less pronounced than during the preoperative phase (p_0–15 dps_ = 0.001, p_15–30 dps_<0.001 and p_30–60 dps_ = 0.001) and became similar to that of the control group. The postoperative visual benefit regarding DVA was more pronounced than the improvement in static visual acuity (p_15 dps_ = 0.001 and p<0.001 at 30 dps, 60 dps and 90 dps).

**Conclusions:**

The impact of age-related cataract on DVA was more severe than its effects on static visual acuity. After cataract surgery, not only static vision of the patients was restored markedly, but also the dynamic vision. DVA could be an important adjunct to the current evaluation system of functional vision, thereby meriting additional attention in clinical assessment.

## Introduction

Dynamic visual acuity (DVA) refers to the ability to resolve a target visually when there is relative motion between the target and the observer [1], [2]. Based on current research, the observer must rely on saccades and smooth pursuits to maintain foveal fixation on the image [1], and the DVA signal is then transmitted by the magnocellular (M) pathway [3], [4]. Relying on these mechanisms, DVA is a relatively independent visual parameter and is different from static visual acuity (SVA) [5] or contrast sensitivity [6], the stimuli of which are captured by high-frequency tremors of the eye [1] and are then transmitted by the parvocellular (P) pathway [7], [8]. Therefore, DVA was identified as a visual measurement meriting additional attention, in a study on vision science by the Committee on Vision [9]. Currently, DVA is mainly applied to investigate visual function related to elite sports performance [10]–[12] and driving safety [13]–[15]. In clinical medicine, DVA has not been widely used.

Owing to its significance in visual hierarchy, dynamic vision is becoming an essential part of studies on the effects of aging on vision. It has been confirmed that there is severe degradation in DVA performance with increasing age after approximately the fourth decade of life [16]–[18]. Moreover, this age-dependent decline in DVA is evident even when elderly participants are prescreened for normal SVA. Population-based investigations have shown that deficiency in visual function related to DVA could contribute to the difficulty in performing of everyday tasks, such as walking [19] and driving [20]. Age-related cataract is a major public health issue and is one of the principal causes of visual decline in the elder [21]. However, the impacts of cataracts on dynamic vision in the elderly and its postoperative rehabilitation have not yet been investigated systemically.

Here, we compared DVA between 26 age-related cataract patients and 30 elderly volunteers without cataract. The changes in DVA in the patient group between the pre- and postoperative phases were also analyzed. The impact of age-related cataract on DVA and its postoperative recovery were discussed intensively. The method used to present a dynamic stimulus was displacement motion, during which the stimulus (optotype) was projected onto a screen and changed its position in the visual field over time [22]. The subjects observed the dynamic optotypes, and DVA was recorded over a series of speed levels. Decreases in velocity-dependent visual acuity were determined by the differences in DVA among consecutive speed levels.

## Methods

### Ethics statement

The research has been approved by Peking University Third Hospital Medicine Ethics Committee and was conducted in accordance with the Declaration of Helsinki. The approval number is IRB00006761-2012046. Written informed consent was obtained from each participant, and the procedures of the entire study were fully explained.

### Participants

The sample size has been calculated by PASS software, version 2008 (NCSS, LLC, Kaysville, UT, USA). Data of 10 cases in the patient group at the very beginning were cited in the calculation. Details of these cases' DVA performances were provided in S1 Table. A paired design was employed (with significance level alpha of 0.05 and power level of 90%) and suggested a minimum sample size of 21 cases. The estimated sample size was 30 cases (about 150% of the minimum sample size).

Thirty (about 150% of 21 cases) consecutive patients with age-related cataract were recruited as the patient group, and finally 26 (12 men and 14 women) patients (mean age: 70.65±1.10 [SEM] years old, range: 59–79) were included in the study (1 patient postponed the cataract surgery, and 3 patients failed to complete the postoperative follow-up). Details of demographic characteristics of the patient group are listed in S1 Table. The cataracts were classified and graded according to LOCS-III criteria [23] under slit-lamp examination. The grading scores were 3.08±0.10 for nuclear color (NC), 3.06±0.10 for nuclear opalescence (NO), 3.21±0.14 for cortical opalescence (C), and 0.96±0.20 for posterior subcapsular opalescence (P), suggesting that cortical and nuclear cataract were the main types of cataract in the current study. Subjects were recruited between July 2011 and May 2013 in the Department of Ophthalmology of Peking University Third Hospital. Inclusion criteria included patients diagnosed of binocular age-related cataract, who had been scheduled for bilateral cataract surgery, were in good general health, had no ocular pathologic features, and experienced no complications during surgery. Exclusion criteria included history of glaucoma or corneal disease, previous ocular surgery or laser treatment, abnormal iris, pupillary deformation, macular degeneration or retinopathy, neuro-ophthalmic disease, and history of vestibular or cerebellar dysfunction. To avoid missing values in data analysis, patients who could not discriminate the largest optotype at any speed level of the DVA test were excluded from the study.

Thirty (14 men and 16 women) elderly volunteers (mean age: 67.97±1.01 [SEM] years old, range: 60–79) who came to our hospital for presbyopic prescription were recruited as the control group. Details of demographic characteristics of the control group are listed in S2 Table. Other than a crystalline lens without cortical or posterior subcapsular opacity in the pupil area and graded as no more than NO-2 or NC-2 by the LOCS-III criteria under slit-lamp examination, the inclusion and exclusion criteria were the same as in the patient group. The control group was comparable to the patient group in terms of gender composition (*χ* ^2^ = 0.001, p = 0.969) and age level (*t* = 1.805, p = 0.077).

Fifty-two eyes of the 26 patients underwent phacoemulsification combined with intraocular lens implantation surgery. The patients were scheduled for clinical evaluations before surgery and at 1 day, 1 week and 1 month after surgery. Postoperative visual acuity was measured 1 month after the bilateral surgery. The elderly volunteers underwent the tests for only one cycle. The subjects underwent the following visual tests with best distance correction. Under uniform regulations, the examinations were performed by rotating residents, who were unaware of objective of the study. To ensure the reliability, a specific recording paper has been designed. In the recording paper, procedures of the measurement were described step by step including the optotype and speed level employed in practice part, the sequence of speed levels in the formal test, how to input speed value at every speed level, how to set the size of the optotype, the sequence of optotypes at every speed level, cycles of every optotype moving on the screen and method to record the results. There was a training for rotating residents participating in the study. Before the formal test, every resident needed to pass a certification exam.

### Surgical procedures

All of the cataract surgeries were performed by one experienced surgeon, who used identical methods for standard phacoemulsification with clear corneal incision, and the spherical monofocal non-yellow-tinted intraocular lenses (IOLs, MatrixAcrylic 401 IOL, Medennium) were inserted through a private injector and cartridge into the capsular bag. The IOL has an overall diameter of 12.5 mm, an optic diameter of 6.0 mm and a refractive index of 1.56. After surgery, the patients were treated with a combination of levofloxacin (Gravit) eye drops and prednisolone acetate (Pred Forte) ophthalmic suspension 4 times per day for 1 week, which were then gradually reduced. There were no intraoperative or postoperative complications in any of the cases.

### Static and dynamic visual acuity tests

Monocular and binocular SVA was measured with the logarithmic visual acuity chart (Precision Vision, La Salle, IL, USA), the optotypes of which were H, O, T and V, at a standard distance of 4 meters.

DVA was examined in a test room specifically designed for evaluating functional vision, and the apparatuses used included a microcomputer running Windows XP software and a projector (Sony, VPL-EX70, Jiangsu Province, China). A custom-designed program was employed to generate dynamic optotypes (the letters H, O, T and V), which were identical to the patterns of the optotypes on the logarithmic visual acuity chart (Precision Vision). Orders were entered using a keyboard to set the size and moving speed of the dynamic optotypes, which were separately projected onto a flat screen. The screen was 1 meter wide and 3 meters high, and it was made of a specific white curtain, which was used for privacy when taking photographs. The subject was seated in front of the screen at a standard distance of 4 meters, and the projector was adjusted to keep the optotype at the eye level of the subject.

Because the purpose of the present study was to discuss functional vision, binocular visual acuity was tested and then included in the following analysis. Speed levels were settled to cover the velocity ranges frequently encountered in daily life [1], [5], [6], [19], [24]. There were four speed levels for the DVA test, including 15, 30, 60 and 90 degree per second (dps). The tests were performed in the order of 90, 60, 30 and 15 dps to avoid the effects of learning. The moving optotype was presented during three cycles for sufficiently long time to detect the optotype. A uniform instruction was given before the test, and practice time was provided to ensure that the subject understood them. In the formal test, there were 2-min breaks between tests at different speeds. The results of the visual acuity tests were recorded in logarithm of the minimum angle of resolution (LogMAR) units.

### Statistical analysis

Statistical analysis was performed using SPSS software, version 16.0 (SPSS, Inc., Chicago, IL, USA). In accordance with previous research [21]–[23], the results are expressed as means ± standard errors of the means (SEMs). The normality of the data distribution was checked by the Kolmogorov-Smirnoff-Lillefors test. Pearson's chi-square test was adopted to analyze the proportions of nominal values. Data on visual acuity were analyzed by Wilcoxon's signed-rank test. The level of statistical significance was p<0.05, and to compare DVA data, the value was 0.017 for Bonferroni's correction.

## Results

### 1. Static visual acuity as a baseline for evaluation

Monocular static visual acuity (SVA) was recorded as a basic index. The compatibility between the SVA of the right eye and of the left eye was analyzed. In the control group, the 30 participants showed similar SVA between the 2 eyes (0.023±0.008 and 0.034±0.012, p = 0.317). There was no statistically significant difference between the monocular SVA of the right eye and the left eye in the patient group (0.485±0.070 and 0.359±0.050, p = 0.166). Postoperatively, the monocular SVA of the right eye and the left eye remained comparable in the patient group (0.021±0.010 and 0.011±0.005, p = 0.203).

The binocular SVA of the patient group was significantly worse than that of the control group (0.194±0.044 compared to 0.006±0.004, p<0.001). After surgery, the patient group exhibited far better binocular SVA (0.002±0.002, p<0.001), which was similar to the control group (p = 0.491).

### 2. Effects of age-related cataract on DVA

**Figure 1:**
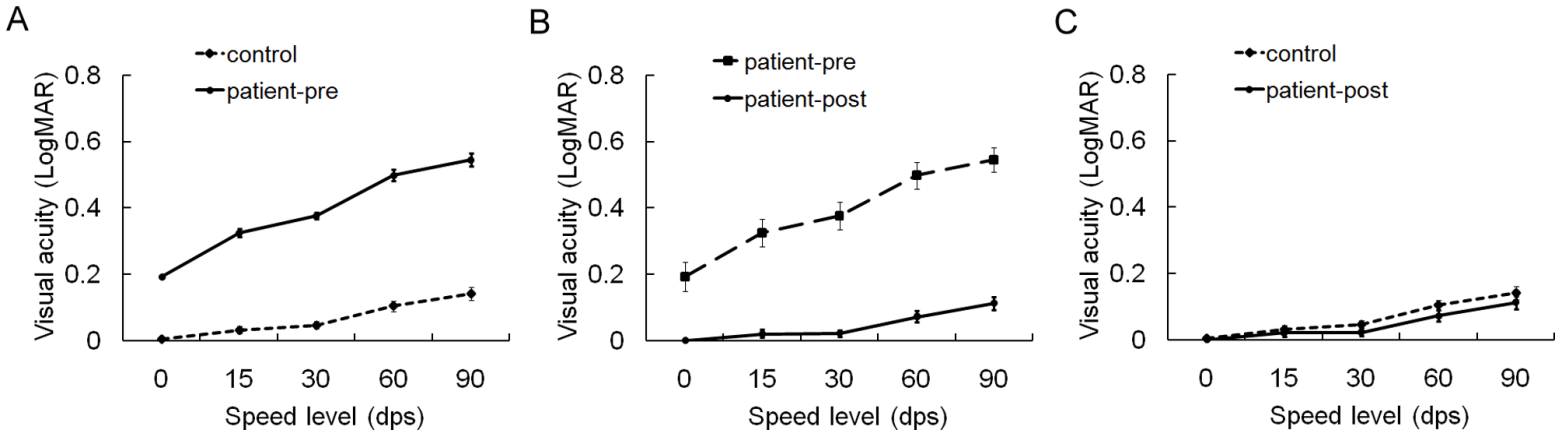
Effects of optotype speed on dynamic visual acuity (DVA). The resolution of dynamic optotypes in the control group and in the patient group was adversely affected by increasing speed. During the preoperative phase, the patient group showed worse DVA performance than the control group at all the four speed levels (p<0.001) (A). Postoperatively, DVA performance in the patient group improved significantly (p<0.001) (B) and recovered to levels comparable to the control group (C). SVA was treated as visual acuity at 0 dps. Error bars depict standard errors of the means (SEMs). LogMAR, logarithm of the minimum angle of resolution; dps, degree per second; pre, preoperative; post, postoperative.

DVA performance in the control group was 0.034±0.010 at 15 dps, 0.048±0.011 at 30 dps, 0.105±0.016 at 60 dps and 0.142±0.020 at 90 dps. The patient group showed worse DVA performance than the control group at all four speed levels (0.325±0.042 at 15 dps, 0.377±0.041 at 30 dps, 0.498±0.040 at 60 dps and 0.545±0.037 at 90 dps, p<0.001; see Table 1 and Fig. 1A). DVA performance of individual participants at specific speed levels is shown in S1 and S2 Tables.

**Table 1:** Binocular static visual acuity and dynamic visual acuity.

Groups	SVA (logMAR)	DVA (logMAR)			
		15 dps	30 dps	60 dps	90 dps
Control group	0.006±0.004	0.034±0.010	0.048±0.011	0.105±0.016	0.143±0.020
Patient group-pre	0.194±0.044*	0.325±0.042*	0.377±0.041*	0.498±0.040*	0.545±0.037*
Patient group-post	0.002±0.002#	0.022±0.012#	0.024±0.010#	0.074±0.017#	0.114±0.019#

Note. SVA, static visual acuity; DVA, dynamic visual acuity; logMAR, logarithm of the minimum angle of resolution; dps, degree per second; pre, preoperative; post, postoperative.

Values are presented as means ± standard errors of the means (SEMs).

*indicates statistical significance when comparing the control group and the patient group during the preoperative phase.

# indicates statistical significance when comparing the pre- and postoperative phases in the patient group.

**Figure 2:**
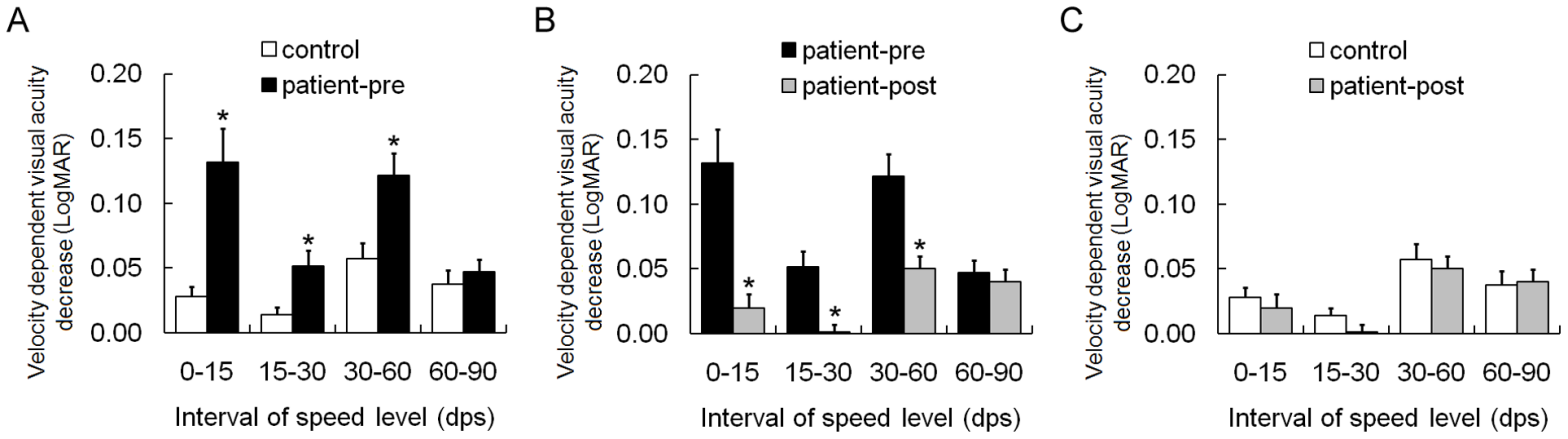
Comparison of decreases in velocity-dependent visual acuity. The patient group experienced more significant decreases in velocity-dependent dynamic visual acuity (DVA) than the control group at the 0–15 dps (p<0.001), 15–30 dps (p = 0.007) and 30–60 dps (p = 0.008) intervals (A). Postoperatively, DVA decline in the patient group was gentler than that during preoperative phase (p≤0.001) (B) and was similar to that of the control group (C). The static condition was treated as a speed level of 0 dps. Error bars depict standard errors of the means (SEMs). LogMAR, logarithm of the minimum angle of resolution; dps, degree per second; pre, preoperative; post, postoperative.

Decreases in velocity-dependent visual acuity, expressed as the difference in DVA between consecutive speed levels, were analyzed to evaluate the degree to which the moving speed interfered with a subject's visual resolution. In the control group, DVA decline was 0.028±0.008 at the interval of 0–15 dps (the static condition was treated as a speed level of 0 dps), 0.014±0.006 at the interval of 15–30 dps, 0.057±0.012 at the interval of 30–60 dps and 0.037±0.011 at the interval of 60–90 dps. In the patient group, DVA decreases at these sequential speed intervals were 0.132±0.026, 0.052±0.012, 0.122±0.017 and 0.047±0.010, respectively. The patient group experienced more significant velocity-dependent DVA decreases than the control group (see Fig. 2A) at the intervals of 0–15 dps (p<0.001), 15–30 dps (p = 0.007) and 30–60 dps (p = 0.008), whereas no significant differences were detected when comparing the patient group and the control group at the 60–90 dps interval (p = 0.509).

### 3. Postoperative recovery of DVA

When the crystalline lens with cataract was replaced by a clear IOL, DVA performance in the patient group (0.022±0.012 at 15 dps, 0.024±0.010 at 30 dps, 0.074±0.017 at 60 dps and 0.114±0.019 at 90 dps) was much better than during the preoperative phase (p<0.001, see Table 1 and Fig. 1B), and it recovered to levels compatible with the control group (p_15 dps_ = 0.254, p_30 dps_ = 0.098, p_60 dps_ = 0.139 and p_90 dps_ = 0.226; see Fig. 1C). Postoperative DVA performance of individual patient at specific speed level is shown in S1 Table.

After bilateral cataract surgery, decreases in velocity-dependent visual acuity in the patient group (0.020±0.011 at the interval of 0–15 dps, 0.002±0.006 at the interval of 15–30 dps, 0.050±0.010 at the interval of 30–60 dps and 0.040±0.010 at the interval of 60–90 dps) were also gentler than during the preoperative phase (p_0–15 dps_ = 0.001, p_15–30 dps_<0.001 and p_30–60 dps_ = 0.001; see Fig. 2B) and were similar to those in the control group (p_0–15 dps_ = 0.326, p_15–30 dps_ = 0.221 and p_30–60 dps_ = 0.802; see Fig. 2C). At the 60–90 dps interval, there were no significant differences in visual decreases, between the pre- and postoperative phases (p = 0.625) or between the postoperative patient group and the control group (p = 0.953).

### 4. Postoperative visual improvement in terms of DVA

**Figure 3:**
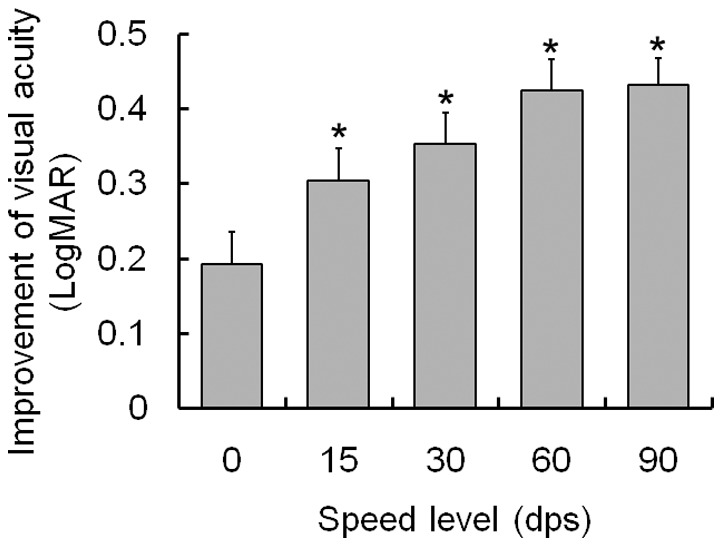
Comparison of postoperative visual benefit between static and dynamic setting. Postoperative improvement in dynamic visual acuity (DVA) at every speed level was more statistically significant than the improvement of binocular static visual acuity (SVA) (p≤0.001). The static condition was treated as a speed level of 0 dps. Error bars represent standard errors of the mean values (SEMs). LogMAR, logarithm of the minimum angle of resolution; dps, degree per second.

In the patient group, postoperative visual improvement at every speed level was manifested by visual acuity changes between the preoperative phase and the postoperative phase. The visual improvement in binocular SVA was 0.192±0.044. Improvements in DVA were 0.303±0.044 at 15 dps, 0.353±0.042 at 30 dps, 0.425±0.041 at 60 dps and 0.432±0.036 at 90 dps. The data suggested that, at all of the speed levels, improvement in DVA was more pronounced than improvement in SVA (p_15 dps_ = 0.001 and p<0.001 at 30 dps, 60 dps and 90 dps; see Fig. 3).

## Discussion

Here, we introduced DVA as a functional parameter for evaluating motion perception in patients with age-related cataracts. The results suggest that the ability to distinguish the fine details of a moving target was intensively affected by cataracts, and this visual interference became more obvious with the increase in speed. Phacoemulsification combined with IOL implantation surgery could restore dynamic vision in the elderly effectively. Postoperative visual benefits were more significant in DVA than those manifested by SVA.

We employed displacement motion to present dynamic optotypes, during which the stimulus with constant elements changed position in the visual field over time [22]. DVA in the control group exhibited a tendency toward a decrease with increasing velocity. The overall pattern was generally consistent with previously reported findings in normal subjects [1], [6], [22], implying the compatibility of our equipment and procedures.

According to theories of dynamic vision, marginal artifacts of the retinal image, which are named retinal smear, have been identified as the cause for decreases in visual acuity with increasing target velocities [1], [25]. With age-related cataract, interaction with light includes occlusive effects and scattering effects, which result in a blurred image on the retina. Retinal smear of a blurred image is much more serious than that of a sharp image [1], and it might explain the significant impact of cataract on DVA. In addition, we believe that serious retinal smear can be treated as background interference to the target awaiting resolution. Studies on aging of vision have confirmed that elderly people are less able to extract visual information in the presence of background interference [26], [27]. Therefore, when subjected to dynamic optotypes, DVA performance in the patient group was more obviously disturbed. Investigators have already noted the discrepancy between results of traditional visual tests and functional visual tests that more closely resemble real-world visual targets [28]. Because dynamic targets are the optotypes that patients need to face and observe in their daily activities, DVA might be a meaningful adjunct to the current evaluation system for functional vision related to senile cataract, and it could become a promising indicator to differentiating patients from normal subjects.

Optical factors have been recognized as one of the reasons for the deterioration of dynamic vision in elderly people [26], [27], [29], [30]. After cataract surgery, the optical characteristics of elderly eyes have been effectively restored. Then, the image on the retina becomes sharper and clearer, resulting in fewer artifacts during movement. Thus, improvement in dynamic visual function is a combination of refined refractive quality, less retinal smear and the elimination of background interference. Additionally, when tracking dynamic targets, the motor system of the eye would evoke saccadic suppression and saccadic omission to avoid excessive perception of blurred images [31], [32]. These suppressions and omissions cause decreases in visual sensitivity to dynamic targets [1]. We expected that the refined retinal image after cataract surgery would induce less saccadic suppression and saccadic omission and thus affect the motor system of the eye positively. DVA has been conceptualized as a parameter that encompasses the optical, sensory, and motor components of the visual system [5], [6]. Therefore, it could detect enhancement of visual function resulting from cataract surgery more effectively. Moreover, dynamic vision was related more directly to the visual status in daily life; therefore, significant improvement in DVA implied a more profound experience of recovered vision than that reflected by traditional tests. A combination of dynamic and static visual evaluations might be an optimal method for assessing postoperative visual outcomes after cataract surgery.

In conclusion, our study provides preliminary evidence to suggest that dynamic visual acuity is an essential supplement to functional vision before and after cataract surgery. DVA might become a promising indicator for its sensitivity in recognizing patients with age-related cataract and manifesting postoperative visual benefits. Limitations of this study include the small sample size, the lack of analysis on the impact of different classifications of cataract on DVA and whether monocular or binocular surgery has an effect on postoperative recovery of dynamic vision. These factors might limit the validity and generalizability of the results. The relationship between DVA and subjective visual quality also needs to be analyzed in future studies. Observations on the ability to resolve dynamic targets against backgrounds simulating daily life could be designed to determine visual rehabilitation in a more practical context.

## Supporting Information

S1 Table **Demographic characteristics and binocular visual acuity of the patient group.**
(DOC)

S2 Table **Demographic characteristics and binocular visual acuity of the control group.**
(DOC)

## References

[1] Geer I, Robertson KM (1993) Measurement of central and peripheral dynamic visual acuity thresholds during ocular pursuit of a moving target. Optom Vis Sci 70:552–560. 8355967 10.1097/00006324-199307000-00006

[2] Erickson GB, Citek K, Cove M, Wilczek J, Linster C, et al (2011) Reliability of a computer-based system for measuring visual performance skills. Optometry 82:528–542. 21705283 10.1016/j.optm.2011.01.012

[3] McKendrick AM, Sampson GP, Walland MJ, Badcock DR (2007) Contrast sensitivity changes due to glaucoma and normal aging: low-spatial-frequency losses in both magnocellular and parvocellular pathways. Invest Ophthalmol Vis Sci 48:2115–2122. 17460269 10.1167/iovs.06-1208

[4] Gilmore GC, Wenk HE, Naylor LA, Stuve TA (1992) Motion perception and aging. Psychol Aging 7:654–660. 1466834 10.1037//0882-7974.7.4.654

[5] Al-Awar Smither J, Kennedy RS (2010) A portable device for the assessment of dynamic visual acuity. Appl Ergon 41:266–273. 19683701 10.1016/j.apergo.2009.07.008

[6] Long GM, May PA (1992) Dynamic visual acuity and contrast sensitivity for static and flickered gratings in a college sample. Optom Vis Sci 69:915–922. 1300512 10.1097/00006324-199212000-00001

[7] Hendry SH, Calkins DJ (1998) Neuronal chemistry and functional organization in the primate visual system. Trends Neurosci 21:344–349. 9720602 10.1016/s0166-2236(98)01245-4

[8] Castelo-Branco M, Faria P, Forjaz V, Kozak LR, Azevedo H (2004) Simultaneous comparison of relative damage to chromatic pathways in ocular hypertension and glaucoma: correlation with clinical measures. Invest Ophthalmol Vis Sci 45:499–505. 14744891 10.1167/iovs.03-0815

[9] Committee on Vision of the National Research Council (US) (1985) Emergent techniques for the assessment of visual performance. Washington: National Academy Press.

[10] Rouse MW, DeLand P, Christian R, Hawley J (1988) A comparison study of dynamic visual acuity between athletes and nonathletes. J Am Optom Assoc 59:946–950. 3209790

[11] Schneiders AG, John Sullivan S, Rathbone EJ, Louise Thayer A, Wallis LM, et al (2010) Visual acuity in young elite motorsport athletes: a preliminary report. Phys Ther Sport 11:47–49. 20381000 10.1016/j.ptsp.2010.01.001

[12] Uchida Y, Kudoh D, Murakami A, Honda M, Kitazawa S (2012) Origins of superior dynamic visual acuity in baseball players: superior eye movements or superior image processing. PLoS One 7:e31530. 22384033 10.1371/journal.pone.0031530 PMC3285166

[13] Shinar D, Schieber F (1991) Visual requirements for safety and mobility of older drivers. Hum Factors 33:507–519. 1769671 10.1177/001872089103300503

[14] Wood JM (2002) Age and visual impairment decrease driving performance as measured on a closed-road circuit. Hum Factors 44:482–494. 12502165 10.1518/0018720024497664

[15] Wilkins L, Gray R, Gaska J, Winterbottom M (2013) Motion perception and driving: predicting performance through testing and shortening braking reaction times through training. Invest Ophthalmol Vis Sci 54:8364–8374. 24282222 10.1167/iovs.13-12774

[16] Burg A (1966) Visual acuity as measured by dynamic and static tests: a comparative evaluation. J Appl Psychol 50:460–466. 5978038 10.1037/h0023982

[17] Ishigaki H, Miyao M (1994) Implications for dynamic visual acuity with changes in aged and sex. Percept Mot Skills 78:363–369. 8022663 10.2466/pms.1994.78.2.363

[18] Long GM, Crambert RF (1990) The nature and basis of age-related changes in dynamic visual acuity. Psychol Aging 5:138–143. 2317293 10.1037//0882-7974.5.1.138

[19] Patel I, Turano KA, Broman AT, Bandeen-Roche K, Munoz B, et al (2006) Measures of visual function and percentage of preferred walking speed in older adults: the Salisbury Eye Evaluation Project. Invest Ophthalmol Vis Sci 47:65–71. 16384945 10.1167/iovs.05-0582

[20] McGregor LN, Chaparro A (2005) Visual difficulties reported by low-vision and nonimpaired older adult drivers. Hum Factors 47:469–478. 16435689 10.1518/001872005774859953

[21] Thylefors B (1990) The World Health Organization's programme for the prevention of blindness. Int Ophthalmol 14:211–219. 2188924 10.1007/BF00158321

[22] Lewis P, Rosen R, Unsbo P, Gustafsson J (2011) Resolution of static and dynamic stimuli in the peripheral visual field. Vision Res 51:1829–1834. 21722661 10.1016/j.visres.2011.06.011

[23] Chylack LT Jr, Wolfe JK, Singer DM, Leske MC, Bullimore MA, et al (1993) The Lens Opacities Classification System III. The Longitudinal Study of Cataract Study Group. Arch Ophthalmol 111:831–836. 8512486 10.1001/archopht.1993.01090060119035

[24] Hoffman LG, Rouse M, Ryan JB (1981) Dynamic visual acuity: a review. J Am Optom Assoc 52:883–887. 7320383

[25] Spering M, Montagnini A (2011) Do we track what we see? Common versus independent processing for motion perception and smooth pursuit eye movements: a review. Vision Res 51:836–852. 20965208 10.1016/j.visres.2010.10.017

[26] Bertone A, Guy J, Faubert J (2011) Assessing spatial perception in aging using an adapted Landolt-C technique. Neuroreport 22:951–955. 22045257 10.1097/WNR.0b013e32834d2f49

[27] Pilz KS, Bennett PJ, Sekuler AB (2010) Effects of aging on biological motion discrimination. Vision Res 50:211–219. 19941881 10.1016/j.visres.2009.11.014

[28] Schneck ME, Haegerstrom-Portnoy G (2003) Practical assessment of vision in the elderly. Ophthalmol Clin North Am 16:269–287. 12809163 10.1016/s0896-1549(03)00008-7

[29] Owsley C (2011) Aging and vision. Vision Res 51:1610–1622. 20974168 10.1016/j.visres.2010.10.020 PMC3049199

[30] Mateus C, Lemos R, Silva MF, Reis A, Fonseca P, et al (2013) Aging of low and high level vision: from chromatic and achromatic contrast sensitivity to local and 3D object motion perception. PLoS One 8:e55348. 23383163 10.1371/journal.pone.0055348 PMC3561289

[31] Campbell FW, Wurtz RH (1978) Saccadic omission: why we do not see a grey-out during a saccadic eye movement. Vision Res 18:1297–1303. 726271 10.1016/0042-6989(78)90219-5

[32] Watson TL, Krekelberg B (2009) The relationship between saccadic suppression and perceptual stability. Curr Biol 19:1040–1043. 19481454 10.1016/j.cub.2009.04.052 PMC3254668

