# Supplementary material for: Significant Improvement in Dynamic Visual Acuity after Cataract Surgery: A Promising Potential Parameter for Functional Vision
Source: PLoS One. 2014 Dec 26;9(12):e115812. doi: 10.1371/journal.pone.0115812 (PMC4277412; doi:10.1371/journal.pone.0115812)
Supplement: S1 Table — Demographic characteristics and binocular visual acuity of the patient group. (DOC) [file pone.0115812.s001.doc]

Table S1. Demographic characteristics and binocular visual acuity of the patient group

| No. | Age  (y.o.d) | Male/  female | Preoperative biocular visual acuity (logMAR) | | | | | Postoperative biocular visual acuity (logMAR) | | | | |
| --- | --- | --- | --- | --- | --- | --- | --- | --- | --- | --- | --- | --- |
|  |  |  | 0  dps | 15  dps | 30  dps | 60  dps | 90  dps | 0  dps | 15  dps | 30  dps | 60  dps | 90  dps |
| 1^﹡^ | 77 | male | 0 | 0.2 | 0.3 | 0.42 | 0.46 | 0 | 0 | 0.04 | 0.12 | 0.16 |
| 2^﹡^ | 76 | male | 0 | 0.24 | 0.32 | 0.46 | 0.46 | 0 | 0 | 0.06 | 0.16 | 0.22 |
| 3^﹡^ | 61 | female | 0.4 | 0.42 | 0.44 | 0.66 | 0.66 | 0 | 0 | 0 | 0 | 0 |
| 4^﹡^ | 78 | female | 0.2 | 0.24 | 0.4 | 0.58 | 0.68 | 0 | 0 | 0 | 0.14 | 0.16 |
| 5^﹡^ | 62 | male | 0 | 0.26 | 0.46 | 0.46 | 0.46 | 0 | 0 | 0.04 | 0.08 | 0.12 |
| 6^﹡^ | 79 | male | 0.46 | 0.46 | 0.62 | 0.64 | 0.72 | 0 | 0 | 0 | 0.02 | 0.08 |
| 7^﹡^ | 74 | female | 0.3 | 0.54 | 0.56 | 0.56 | 0.6 | 0 | 0 | 0.02 | 0.12 | 0.18 |
| 8^﹡^ | 79 | male | 0.1 | 0.26 | 0.3 | 0.5 | 0.54 | 0.04 | 0.12 | 0.02 | 0.14 | 0.2 |
| 9^﹡^ | 73 | female | 0 | 0.02 | 0.02 | 0.18 | 0.2 | 0 | 0 | 0 | 0 | 0 |
| 10^﹡^ | 66 | female | 0.34 | 0.3 | 0.3 | 0.3 | 0.46 | 0 | 0 | 0 | 0 | 0 |
| 11 | 69 | male | 0.02 | 0.1 | 0.16 | 0.2 | 0.24 | 0 | 0 | 0 | 0.06 | 0.08 |
| 12 | 72 | female | 0 | 0.24 | 0.4 | 0.54 | 0.62 | 0 | 0 | 0 | 0 | 0 |
| 13 | 76 | female | 0.18 | 0.3 | 0.3 | 0.46 | 0.5 | 0 | 0 | 0 | 0.1 | 0.16 |
| 14 | 71 | female | 0 | 0.18 | 0.22 | 0.34 | 0.44 | 0 | 0 | 0 | 0 | 0.02 |
| 15 | 59 | female | 0.4 | 0.34 | 0.4 | 0.6 | 0.6 | 0.02 | 0.28 | 0.24 | 0.4 | 0.4 |
| 16 | 66 | female | 0.1 | 0.2 | 0.22 | 0.42 | 0.46 | 0 | 0.02 | 0.02 | 0.02 | 0.1 |
| 17 | 76 | male | 0.2 | 0.36 | 0.4 | 0.6 | 0.6 | 0 | 0.1 | 0.08 | 0.1 | 0.1 |
| 18 | 67 | female | 0 | 0.2 | 0.2 | 0.24 | 0.3 | 0 | 0 | 0 | 0 | 0 |
| 19 | 68 | female | 0 | 0.16 | 0.18 | 0.46 | 0.5 | 0 | 0 | 0.04 | 0.12 | 0.14 |
| 20 | 71 | male | 1 | 1 | 1 | 1 | 1 | 0 | 0 | 0.02 | 0.02 | 0.16 |
| 21 | 73 | male | 0.2 | 0.26 | 0.32 | 0.6 | 0.58 | 0 | 0 | 0 | 0.08 | 0.1 |
| 22 | 64 | male | 0.3 | 0.32 | 0.32 | 0.46 | 0.52 | 0 | 0 | 0 | 0 | 0.2 |
| 23 | 73 | male | 0.3 | 0.3 | 0.4 | 0.44 | 0.62 | 0 | 0.02 | 0.04 | 0.14 | 0.2 |
| 24 | 67 | female | 0.3 | 0.86 | 0.86 | 1 | 1 | 0 | 0 | 0 | 0.06 | 0.16 |
| 25 | 69 | female | 0.24 | 0.5 | 0.5 | 0.62 | 0.66 | 0 | 0.02 | 0 | 0 | 0 |
| 26 | 71 | male | 0 | 0.2 | 0.2 | 0.22 | 0.3 | 0 | 0.02 | 0 | 0.04 | 0.02 |

Note. y.o.d=years of old; logMAR=logarithm of the minimum angle of resolution; dps=degree per second. The static condition was treated as a speed level of 0 dps.

^﹡^indicates cases that were cited in the sample size calculation.
